# Determination of p53 genotypes in oral cancer patients from India

**DOI:** 10.1054/bjoc.2000.1674

**Published:** 2001-03

**Authors:** A T Tandle, V Sanghvi, D Saranath

**Affiliations:** Laboratory of Cancer Genes, Cancer Research Institute; Tata Memorial Hospital, Tata Memorial Centre, Parel, Mumbai –, 400 012, India

**Keywords:** oral cancer, *p53* gene, codon 72 polymorphism, loss of heterozygosity

## Abstract

The *p53* tumour suppressor gene is inactivated in various types of human cancers, and has been implicated as an early event in several cancers. A p53 Pro/Arg polymorphism at exon 4 codon 72, has been suggested to be involved in susceptibility to cancers as well. Hence, in the current study, we investigated p53 exon 4 codon 72 polymorphism using Proline or Arginine specific primers from the peripheral blood cells (PBC) representing constitutional DNA from 72 oral cancer patients. PBC from 153 normal healthy individuals were used to determine the frequency of the p53 genotypes, Pro/Pro, Arg/Arg and Pro/Arg, in the Indian population. The frequency of distribution of genotypes in the normal healthy individuals was, Pro/Pro – 0.20 (31/153), Arg/Arg – 0.14 (22/153) and Pro/Arg – 0.65 (100/153); and in the oral cancer patients was, Pro/Pro – 0.19 (14/72), Arg/Arg – 0.08 (6/72) and Pro/Arg – 0.72 (52/72). Thus, we observed an equidistribution of the genotypes in normal control and oral cancer patients (χ^2^= 1.77, df = 2, 0.3 <*P*< 0.5). Further, DNA from corresponding tumours from the 72 oral cancer patients were examined for loss of heterozygosity in the *p53* gene. Allelic loss was observed in 8 of 52 (15%) heterozygous informative oral cancer patients. Our data indicates an equidistribution of the genotypes and absence of over-representation of either Pro/Pro or Arg/Arg genotypes in the oral cancer patients as compared to the normal healthy controls. Hence, association of the p53 genotypes with susceptibility to oral cancer, was not observed. © 2001 Cancer Research Campaign http://www.bjcancer.com


British Journal of Cancer (2001) **84**(6), 739–742
© 2001 Cancer Research Campaign
doi: 10.1054/ bjoc.2000.1674, available online at http://www.idealibrary.com on IDEAL®

### Determination of p53 genotypes in oral cancer patients from India

**AT Tandle[1], V Sanghvi[2] and D Saranath[1]**

[1]Laboratory of Cancer Genes, Cancer Research Institute; [2]Tata Memorial Hospital, Tata Memorial Centre, Parel, Mumbai – 400 012, India

**Summary** The *p53* tumour suppressor gene is inactivated in various types of human cancers, and has been implicated as an early event in several cancers. A p53 Pro/Arg polymorphism at exon 4 codon 72, has been suggested to be involved in susceptibility to cancers as well. Hence, in the current study, we investigated p53 exon 4 codon 72 polymorphism using Proline or Arginine specific primers from the peripheral blood cells (PBC) representing constitutional DNA from 72 oral cancer patients. PBC from 153 normal healthy individuals were used to determine the frequency of the p53 genotypes, Pro/Pro, Arg/Arg and Pro/Arg, in the Indian population. The frequency of distribution of genotypes in the normal healthy individuals was, Pro/Pro – 0.20 (31/153), Arg/Arg – 0.14 (22/153) and Pro/Arg – 0.65 (100/153); and in the oral cancer patients was, Pro/Pro – 0.19 (14/72), Arg/Arg – 0.08 (6/72) and Pro/Arg – 0.72 (52/72). Thus, we observed an equidistribution of the genotypes in normal control and oral cancer patients ($\chi^2$ = 1.77, df = 2, 0.3 < *P* < 0.5). Further, DNA from corresponding tumours from the 72 oral cancer patients were examined for loss of heterozygosity in the *p53* gene. Allelic loss was observed in 8 of 52 (15%) heterozygous informative oral cancer patients. Our data indicates an equidistribution of the genotypes and absence of over-representation of either Pro/Pro or Arg/Arg genotypes in the oral cancer patients as compared to the normal healthy controls. Hence, association of the p53 genotypes with susceptibility to oral cancer, was not observed. © 2001 Cancer Research Campaign http://www.bjcancer.com

**Keywords**: oral cancer; *p53* gene; codon 72 polymorphism; loss of heterozygosity

Oral cancers represent 4% of all cancers in the West, whereas, in developing countries including India, it accounts for up to 45% of all cancers (Johnson and Warnakulasuriya, 1993). Globally, oral cancer ranks as the sixth most common cancer, with evidence of an increase in incidence rate and mortality due to this cancer in recent years, particularly in young adults in Central Europe (Notani, 2000). The major aetiologic factors in the development of oral cancer constitute tobacco chewing/smoking and alcohol consumption (Choi and Kahyo, 1991; Johnson and Warnakulasuriya, 1993; Notani, 2000). Recently high-risk human papillomaviruses (HPVs), have been implicated as important aetiologic agents, particularly in the oral cancers with no tobacco or alcohol associations (D'Costa et al, 1998; Gillison et al, 2000). In spite of advances in surgery, radiotherapy and chemotherapy, the 5-year survival rates in oral cancer are among the lowest (Johnson, 1991). In India, greater than 70% of oral cancers are observed in advanced stages III/IV, by the clinicians. Unlike in the West, most of the oral cancers seen in India, are preceded by distinct premalignant lesions such as leukoplakia and submucous fibrosis (Gupta et al, 1989). Further, although a majority of oral cancers occur in individuals with tobacco/alcohol habits, only a small proportion of tobacco habits develop oral cancer. Hence, identification of high-risk premalignant lesions with increased susceptibility to oral cancer and consequent aggressive follow up for change of habit and early detection, may help in down staging of the cancer and better prognosis. A genetic marker to indicate increased susceptibility would be useful in this scenario.

Studies from our laboratory on molecular alterations including genomic instability using microsatellite markers and inactivation of *p53* tumour suppressor gene in oral cancers and oral lesions have been reported (Saranath et al, 1999; Mahale and Saranath, 2000). The *p53* gene inactivation via mutations, overexpression, deletions and binding to viral proteins has been demonstrated in oral cancers and oral lesions (Raybaud-Diogene et al, 1996; Saranath et al, 1997, 1999). Recently, a common polymorphism in *p53* gene at exon 4 codon 72, encoding for either a proline (CCC) or arginine (CGC) amino acid, has been implicated in susceptibility to certain cancers, with a reported increased prevalence of Arg/Arg genotype in cervical cancer and Pro/Pro genotype in lung cancer (Kawajiri et al, 1993; Jin et al, 1995; Murata et al, 1996; Storey et al, 1998). On the other hand, no association between either Pro/Pro or Arg/Arg genotypes and increased susceptibility has been reported for cancers of breast, bladder, colorectal and head and neck (H & N) (Kawajiri et al, 1993; Hamel et al, 2000).

In the present study we analysed p53 genotypes in oral cancer patients, to examine the genotypic distribution and association with susceptibility to the cancer. The p53 polymorphism was studied in constitutional peripheral blood cell (PBC) DNA, both from the cancer patients and from normal controls. This was essential, as tumour associated lesions in the p53 polymorphic loci, may introduce a bias in genotypic analysis. Further, in our previous studies examining loss of heterozygosity (LOH) in oral cancer tissues, using the same p53 polymorphism in a PCR-restriction fragment length polymorphism (PCR-RFLP) assay, tumour-associated LOH was observed in 20% of the samples (Saranath et al, 1997). The earlier study used primers to amplify exon 4 codon 72, with the PCR product digested by AccII enzyme,

*Received 31 August 2000*
*Revised 22 November 2000*
*Accepted 20 December 2000*

*Correspondence to:* D Saranath to examine tumour-associated LOH. However, the genotypic distribution was not analysed in the study.

### MATERIALS AND METHODS

### Study population

The samples constituted 72 oral cancer patients, with histopathologically confirmed squamous cell carcinoma (SCC) of the oral cavity. Primary site of tumours included buccal mucosa – 39 cases, alveolus – 22 cases, tongue – 10 cases and lip – 1 case. 20 ml peripheral blood and matching primary tumours were collected from all the patients at the time of surgery, from Tata Memorial Hospital, Mumbai, India. Informed consent was obtained from the patients. Tumour tissues and the leucocytes separated from the peripheral blood, were stored at –70˚C until further use.

Peripheral blood cells from 153 normal healthy volunteers were collected to compare the p53 codon 72 polymorphism in cancer patients with that in healthy individuals of Indian ethnicity.

### DNA extraction

DNA was extracted from the PBCs and oral tumour tissue samples using standard procedure, involving SDS/Proteinase K, ribonuclease A, phenol-chloroform extraction of proteins and ethanol precipitation of DNA, as detailed earlier (Saranath et al, 1989).

### PCR

Analysis of the p53 genotype at exon 4 codon 72, was performed as described by Storey et al (1998), with minor modifications. Proline and arginine specific sequences were used for the in vitro amplification. The primer sequences were as follows: Pro specific primers: Sense primer (codons 66–72): 5′ GCC AGA GGC TGC TCC CCC 3′, Antisense primer (Codons 120–126): 5′ CGT GCA AGT CAC AGA CTT 3′; Arg specific primers: Sense primer (Codons 33–38): 5′ TCC CCC TTG CCG TCC CAA 3′, Antisense primer (Codons 72–78): 5′ CTG GTG CAG GGG CCA CGC 3′. Briefly, the reaction mixture contained 100 ng of sample DNA, in a total reaction volume of 15 µl constituting 10 mM Tris buffer pH 8.4, 50 mM KCl, 1.5 mM $MgCl_2$; 200 µM each of dATP, dCTP, dGTP, and dTTP; 100 ng each Pro sense and antisense primers and a duplicate tube with Arg sense and antisense primers; 0.01% bovine serum albumin and 0.125 units of Taq polymerase (Gibco-BRL, USA). The DNA was subjected to 35 cycles of PCR with denaturation at 94˚C for 30 s, annealing at 59˚C for 30 s, and extension at 72˚C for 30 s, followed by a final extension at 72˚C for 5 min.

The PCR products were electrophoresed on 11% denaturing polyacryamide gel, at 50˚C, using 25 mA current for 2 h. After electrophoresis the gel was fixed in 10% acetic acid: 45% methanol for 30 min, and subjected to two washes of 50% ethanol. The p53 alleles were visualized by silver staining. Briefly, the gel was treated with 3% nitric acid, rinsed with distilled water and treated with 0.2% silver nitrate in presence of 0.1% formaldehyde. The bands were visualized by treating the gel with 3% sodium carbonate containing 0.1% formaldehyde, until a strong brown colour was apparent. The reaction was arrested by immersing the gel in 2% glacial acetic acid.

### Statistical analysis

Chi-square test was performed to compare p53 genotype distribution in the patient and control groups. The Hardy–Weinberg equilibrium was tested by a goodness-of-fit $\chi^2$ test to compare the observed genotypic frequencies in normal individuals to the expected genotypic frequencies, calculated from the observed allelic frequencies.

### RESULTS

### p53 Pro and Arg genotype frequencies

Polymerase chain reaction amplification using p53 Pro specific primers, amplified a 177 bp fragment; whereas p53 Arg allele demonstrated a 141 bp fragment. A representative polyacrylamide gel with samples showing the different alleles, is demonstrated in

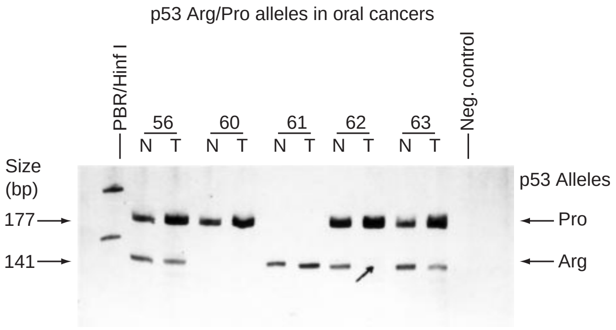

**Figure 1** Polyacrylamide gel electrophoresis of p53 codon 72 alleles in matched normal (N) and oral tumour (T). Pro and Arg specific primers amplified 177 bp and 141 bp products respectively. Pro/Pro homozygous genotype showed 177 bp fragment whereas Arg/Arg homozygous genotype showed 141 bp fragment. Pro/Arg heterozygous genotype demonstrated presence of both the fragments, 177 bp and 141 bp. Negative control (Neg. control) does not contain DNA. Sample 62 shows loss of Arg allele in the tumour tissue, on comparison with corresponding peripheral blood cell DNA

**Table 1** Frequency of *Pro/Arg polymorphism at *p53* gene loci in oral cancer cases and controls and LOH observed in oral cancers

| Samples | p53 Genotypes | | | No. of cases with LOH/No. of heterozygous cases (Pro/Arg) |
|---|---|---|---|---|
| | Pro/Pro *n* (freq) | Arg/Arg *n* (freq) | Pro/Arg *n* (freq) | |
| Oral cancers (*n* = 72) | 14 (0.19) | 6 (0.08) | 52 (0.72) | 8/52 (15) |
| Healthy controls (*n* = 153) | 31 (0.20) | 22 (0.14) | 100 (0.65) | |

*The constitutive presence of Pro/Arg genotypes in the PBC is given. LOH = loss of heterozygosity.

 © 2001 Cancer Research Campaign the figure (Figure 1). Thus, homozygous Pro alleles demonstrated a 177 bp fragment, and homozygous Arg alleles showed 141 bp fragment; whereas p53 Pro/Arg heterozygous alleles showed presence of both the fragments, 177 bp and 141 bp (Figure 1). The Pro/Arg polymorphic frequency distribution in healthy controls and oral cancer patients is shown in Table 1. There was no significant difference in the distribution of the 3 genotypes between the oral cancer patient group and normal healthy controls ($\chi^2 = 1.77$; df = 2; $0.3 < P < 0.5$).

Loss of heterozygosity was studied by comparison of the p53 genotype in the PBC with the observed genotype in corresponding tumour tissue. The loss was detectable as complete loss of an allelic fragment and represented as LOH in the heterozygous samples. LOH was indicated as loss of either the Pro allele or Arg allele. We observed tumour-associated loss in 8 of 52 (15%) heterozygous oral cancer patients as shown in Table 1 (Figure 1).

### Statistical analysis

There was no statistically significant difference in the frequencies of the 3 genotypes Pro/Pro, Arg/Arg and Pro/Arg, between healthy controls and oral cancer patients ($\chi^2 = 1.77$; df = 2; $0.3 < P < 0.5$). However, a significant deviation from the Hardy–Weinberg equilibrium was observed in normal controls ($\chi^2 = 14.775$, df = 1, $P < 0.001$) as well as in oral cancer patients ($\chi^2 = 19.489$, df = 1, $P < 0.001$).

### DISCUSSION

Several studies have examined frequency of the p53 genotypes, Pro/Pro and Arg/Arg in human cancers, such as lung, colorectal, breast, stomach, bladder, H & N and cervical cancer to analyse association of this polymorphism with cancer susceptibility (Kawajiri et al, 1993; Jin et al, 1995; Storey et al, 1998; Hamel et al, 2000). Studies on bladder, breast and colorectal cancer showed no association between p53 polymorphism and the malignancies (Kawajiri et al, 1993; Sjalander et al, 1995). However, presence of Arg/Arg genotype has been associated with increased susceptibility to cervical cancer and Pro/Pro genotype with lung cancers (Kawajiri et al, 1993; Jin et al, 1995; Murata et al, 1996; Storey et al, 1998). A single study on H & N cancer analysing 163 patients from Canada, demonstrated no over-representation of Arg/Arg genotype in H & N SCC cases compared to controls, over different tobacco exposure strata (Hamel et al, 2000).

In the present study, we examined p53 exon 4 codon 72 polymorphism in normal healthy individuals and oral cancer patients, both groups representing ethnically similar backgrounds. PBC DNA was used in both the groups for the analysis, to represent the constitutional genotypes, as tumour-associated lesion in the *p53* gene are common and may introduce a bias in the genotypic frequencies (Saranath et al, 1999). In our studies, the genotypic distribution was similar in the normal individuals and the oral cancer patients ($\chi^2 = 1.77$, df = 2, $0.3 < P < 0.5$), although deviation from Hardy–Weinberg equilibrium was noted in both the control and the oral cancer population. A deviation from Hardy–Weinberg equilibrium among African-American sample controls with respect to the p53 Pro/Arg genotype has been reported earlier, by Jin et al (1995). The authors attributed the deviation to a relative excess of heterozygotes. In this study, besides the excess of heterozygotes in both the controls and the oral cancer patients, the cases as well as controls consisted of non-communicating stratas i.e. Hindus, Muslims, Christians and their subcastes, which generally do not intermarry. Thus, even though Hardy–Weinberg equilibrium holds within a strata, it may not hold in a mixed sample such as the present data. Hamel et al (2000), have indicated the importance of using controls derived from the same population as the cases and adequate sizing of the control group, of crucial importance to the validity of genotyping studies. In the current study, the control and the patient group contained ethnically similar proportion of different castes and subcastes of Indians. Our results in oral cancer showed no over-representation of Arg/Arg genotype in oral cancer patients, analysed in PBC DNA representing the constitutive presence of genotypes. Thus, p53 codon 72 polymorphism was not associated with susceptibility to oral cancer.

In the oral cancer group, we also examined tumour-associated alterations in the p53 gene, by examining corresponding PBC and tumour tissue DNA from individual patients. This enabled us to determine LOH in cancer patients, as allelic deletion is one of the mechanisms of p53 inactivation. Loss of heterozygosity was observed in the current study in 15% of oral cancer tissues. The results were comparable to our earlier study analysing LOH using PCR-RFLP analysis, with 20% of the oral cancer patient tissues showing LOH of p53 exon 4 codon 72 (Saranath et al, 1997). Largey and co-workers (1993) have demonstrated relatively higher LOH of 71% (10/14) in smoking tobacco-associated oral cancers from USA, albeit the sample size of 14 informative cases was rather small. Differences in the molecular profile between Indian chewing tobacco-associated oral cancers and smoking-related cancers from the West have been reported earlier (Saranath et al, 1993, 1999; Raybaud-Diogene et al, 1996; Saranath, 2000).

In the Indian oral cancer patients, the prevalence of chewing tobacco is high, with the attributable risk of tobacco varying from 61% to 70% for the cancer (Notani, 2000). However, not all tobacco habitues develop oral cancer. Genetic susceptibility may give us a clue as to the tobacco habitues who will develop oral cancer with increased possibility. Hence, such studies at specific loci suspected to increase genetic susceptibility to human cancers are necessary. In the current study we observed a difference in the p53 genotypic distribution from reported literature, and an equidistribution of the genotypes in control and oral cancer patients. Thus, p53 genotype was not associated with increased susceptibility to oral cancer.

### ACKNOWLEDGEMENTS

We acknowledge Ms Perin Notani, Consultant Epidemiologist and Biostatistician, Tata Memorial Hospital, Mumbai, for her help in statistical analysis and for critical reading of the manuscript.

### REFERENCES

Choi SY and Kahyo H (1991) Effect of cigarette smoking and alcohol consumption in the aetiology of cancer of the oral cavity, pharynx and larynx. *Int J Epidemiol* **20**: 878–885

D'Costa JD, Saranath D, Dedhia P, Sanghavi V and Mehta AR (1998) Detection of HPV-16 genome in human oral cancers and potentially malignant lesions from India. *Oral Oncol* **34**: 413–420

Gillison ML, Koch WM, Capone RB, Spafford M, Westra WH, Wu L, Zahurak ML, Daniel RW, Viglione M, Symer DE, Shah KV and Sidransky D (2000) Evidence for a casual association between human papillomavirus and a subset of head and neck cancers. *J Natl Cancer Inst* **92**: 709–720

Gupta PC, Bhonsle RB, Murti PR, Daftary DK, Mehta FS and Pindborg JJ (1989) An epidemiologic assessment of cancer risk in oral precancerous lesions in India with special reference to nodular leukoplakia. *Cancer* **63**: 2247–2252

© 2001 Cancer Research Campaign 

Hamel N, Black MJ, Ghadirian P and Foulkes WD (2000) No association between p53 codon 72 polymorphism and risk of squamous cell carcinoma of the head and neck. *Br J Cancer* **82**: 757–759

Jin X, Wu X, Roth JA, Amos CI, King TM, Branch C, Honn SE and Spitz MR (1995) Higher lung cancer risk for African-Americans with the Pro/Pro p53 genotype. *Carcinogenesis* **16**: 2205–2208

Johnson NW (1991) A global view of the epidemiology of oral cancer. In: Johnson NW (ed) *Oral cancer detection of patients and lesions at risk*, Vol 2, pp 3–26. Cambridge University Press: Cambridge

Johnson NW and Warnakulasuriya KAAS (1993) Epidemiology and aetiology of oral cancer in the United Kingdom. *Community Dental Health* **10**: 13–29

Kawajiri K, Nakachi K, Imai K, Watanabe J and Hayashi S (1993) Germ line polymorphisms of p53 and CYP1A1 genes involved in human lung cancer. *Carcinogenesis* **14**: 1085–1089

Largey JS, Meltzer SJ, Yin J, Norris K, Sauk JJ and Archibald DW (1993) Loss of heterozygosity of p53 in oral cancers demonstrated by the polymerase chain reaction. *Cancer* **71**: 1933–1937

Mahale A and Saranath D (2000) Microsatellite alterations on chromosome 9 in chewing-tobacco induced oral squamous cell carcinomas from India. *Oral Oncol* **36**: 199–206

Murata M, Tagawa M, Kimura M, Kimura E, Watanabe S and Saisho H (1996) Analysis of a germ line polymorphism of the p53 gene in lung cancer patients; discrete results with smoking history. *Carcinogenesis* **17**: 261–264

Notani PN (2000) Epidemiology and prevention of head and neck cancer: A global view. In: Saranath D (ed) *Contemporary Issues In Oral Cancer*, pp 1–29. Oxford University Press: New Delhi

Raybaud-Diogene H, Tetu B, Morency R, Fortin A and Monteil RA (1996) p53 overexpression in head and neck squamous cell carcinoma: review of the literature. *Oral Oncol Eur J Cancer* **32B**: 143–149

Saranath D (2000) Integrated biology and molecular pathology of oral cancer. In: Saranath D (ed) Contemporary Issues in Oral Cancer pp 30–71. Oxford University Press: New Delhi

Saranath D, Panchal R, Nair R, Mehta A, Sanghavi V, Sumegi J, Klein G and Deo M (1989) Oncogene amplification in squamous cell carcinoma of the oral cavity. *Jpn J Cancer Res* **80**: 430–437

Saranath D, Bhoite LT and Deo MG (1993) Molecular lesions in human oral cancer: the Indian scene. *Oral Oncol Eur J Cancer* **29B**: 107–112

Saranath D, Tandle AT and Deo MG (1997) Loss of p53 gene as a biomarker of high risk oral leukoplakias. *Ind J Biochem Biophys* **34**: 266–273

Saranath D, Tandle AT, Teni TR, Dedhia PM, Borges AM, Parikh D, Shanghavi V and Mehta AR (1999) p53 inactivation in chewing tobacco-induced oral cancers and leukoplakias from India. *Oral Oncol* **35**: 242–250

Sjalander A, Birgander R, Athlin L, Stenling R, Rutegard J, Beckman L and Beckman G (1995) p53 germ line haplotypes associated with increased risk for colorectal cancer. *Carcinogenesis* **16**: 1461–1464

Storey A, Thomas M, Kalita A, Harwood C, Gardiol D, Mantovani F, Breuer J, Leigh IM, Matlashewski G and Banks L (1998) Role of p53 polymorphism in the development of human papillomavirus-associated cancer. *Nature* **393**: 229–234

*© 2001 Cancer Research Campaign*
